# Establishment of a diagnostic model of coronary heart disease for patients with diabetic foot

**DOI:** 10.1097/MD.0000000000022334

**Published:** 2020-10-02

**Authors:** Ying Chen, Liwei Meng, Liangchen Wang, Li Xiao, Caizhe Yang

**Affiliations:** a Department of Endocrinology of the Air Force Medical Center, PLA; b Department of Endocrinology of the Xiangyang District People's Hospital of Xiangyang City, China.

**Keywords:** chronic renal insufficiency, coronary heart disease, diabetic foot, diagnosis, risk factors, toe-brachial

## Abstract

This study aims to establish a diagnostic model of coronary heart disease (CHD) for diabetic foot (DF) patients.

The clinical data of 489 hospitalized patients with DF were retrospectively analyzed in this case-control study. The patients were divided into the CHD group (DF with CHD, n = 212) and the control group (DF without CHD, n = 277). Univariate analysis was performed to screen for CHD-related risk factors, and multivariate logistic regression analysis was conducted to determine significant CHD risk factors. Scores were assigned according to the ratio of risk factors (OR) to establish a diagnostic model of CHD for patients with DF. The area under the ROC curve was used to test the application value of the diagnostic model.

The logistic regression analysis showed that the risk factors for CHD in DF patients were age, duration of diabetes, toe-brachial index, hyperuricemia, and chronic renal insufficiency. The area under the ROC curve of the diagnostic model was 0.798 (0.759–0.837), the diagnostic point of CHD was 6 points, the diagnostic sensitivity was 69.3%, and the specificity was 76.5%.

The established model has good diagnostic value and provides the basis for preliminary screening for CHD in patients with DF.

## 1 Introduction

The increasing number of people with diabetes has become a global problem. The global prevalence of diabetes reached 8.8% in 2017 and will increase to 9.9% by 2045.^[1]^ The current diabetes rate in China is 11.6%.^[2]^ Diabetic foot (DF) is one of the most common and serious complications of diabetes. The 5-year mortality rate of patients with DF is about 50%.[^3^,^4^,^5^] The major cause of death is coronary heart disease (CHD),[^3^,^6^,^7^] and the risk of sudden death is high. Therefore, screening for CHD in patients with DF is important. Coronary angiography is the gold standard for diagnosis of CHD,^[8]^ Computed tomographic coronary angiography also exhibits high specificity and sensitivity,[^9^,^10^] but is expensive and limited by renal function, so it cannot be used as routine examination for clinical screening of CHD. Routine examination methods for screening CHD include resting electrocardiogram and exercise plate experiment. Resting electrocardiogram has a low positive rate. In particular, the electrocardiogram performance of diabetic patients is often atypical, which leads to misdiagnosis. Exercise plate experiment cannot complete the examination due to foot ulcers. Therefore, establishing a diagnostic model of CHD for patients with DF is of significance.

In this study, the clinical data of patients with DF were retrospectively analyzed to screen for the risk factors of CHD and establish a diagnostic model.

## 2 Methods

### 2.1 Study population

A retrospective analysis was performed on 489 patients with DF hospitalized in the endocrinology department of the Air Force Medical Center, PLA from December 2014 to April 2019. The participants included 366 males and 123 females aged between 31 to 88 years and mean (60.71 ± 10.92) years. All the included subjects met the definition of DF established by the World Health Organization in 1999: “In patients with diabetes mellitus, infection, ulceration, and/or deep tissue destruction of the lower extremity are caused by the concomitant neuropathy and various degrees of vascular diseases”^[11]^ The exclusion criteria were as follows:

(1) complication with obvious life-threatening diseases (such as acute complications of diabetes, tumor, multiple organ failure, etc;
(2) patients who are unable to complete relevant examinations; and
(3) pregnant and lactating women.

CHD was diagnosed with any of the following^[12]^:

(1) electrocardiogram indicating the presence of old myocardial infarction;
(2) who had a history of underwent percutaneous coronary intervention or coronary artery bypass grafting; and
(3) coronary angiography or coronary CT (coronary stenosis ≥ 50%).

Patients with DF were divided into CHD (212 cases) or control (277 cases) group according to the presence or absence of CHD.

### 2.2 Data collection

The following clinical data of the patients were retrospectively analyzed: gender, age, body mass index (BMI), smoking history, duration of diabetes mellitus, glycosylated hemoglobin, fibrinogen, total cholesterol, triglyceride, high density lipoprotein-cholesterol, low density lipoprotein-cholesterol, toe-brachial index (TBI), hypertension, hyperuricemia, and chronic renal insufficiency.

Individuals with BMI ≥ 25 kg/m^2^ were defined as overweight.

Definition of chronic renal insufficiency: according to “2012 Clinical Practice Guideline for the Evaluation and Management of Chronic Kidney Disease” chronic kidney disease staging. Renal insufficiency is defined as glomerular filtration rate <60 mL/ (min•1.73 m^2^).^[13]^

BP-203RPE III network arteriosclerosis detection device (Japan Omron Company) was used to measure TBI. TBI > 0.7 is normal, and TBI ≤ 0.7 indicates obvious lower limb artery stenosis.[^14^,^15^,^16^] Low values of TBI on both sides were selected for analysis.

Diagnosis model establishment: The risk factors of CHD were screened by single-factor analysis, and further evaluated by multiple logistic regression test. The odds ratio (OR) gives scores as per the risk factors, respectively, and the value is rounded to the sum of various risk factors of patients with total score.

Determination of diagnostic points and evaluation of the diagnostic value of the model

Diagnostic points were selected through the receiver operating characteristic (ROC) curve, and their application value was determined through the area under the curve. The area under the ROC curve is between 1.0 and 0.5. When the area under the curve is >0.5, the closer it is to 1, the better the diagnosis effect is. When the area is between 0.5 and 0.7, the accuracy is low; and when the area is 0.7 to 0.9, the accuracy is high. When the area is below 0.5, the diagnosis method is completely ineffective.

### 2.3 Statistical approach

SPSS 22.0 software was used for statistical analysis. Counting data were expressed as a percentage. Comparison between the two groups was conducted by *χ* ^2^ test. X ± s was used to describe the measurement data that met the normal distribution. *T* test was used for comparison between the groups. Nonnormal distribution was described by median (quartile spacing), and comparison between the groups was performed by *U* test. Risk factors for CHD were selected. Multivariate logistic regression was used to determine the OR value of CHD risk factors. The diagnostic point was selected by the ROC curve, and the diagnostic value of the diagnostic model was tested by the area under the ROC curve. *P* < 0.05 was considered statistically significant.

## 3 Results

### 3.1 Comparison of baseline data of the 2 groups

The age of the CHD group was higher than that of the control group (*P* = 0.00). The number of participants with age ≥ 60 years, duration of diabetes ≥ 10 years, TBI ≤ 0.7, chronic renal insufficiency, high blood pressure, and hyperuricemia, was higher in the CHD group than the control group (*P* = 0.00). The proportion of patients with BMI ≥ 25 was higher in the CHD group than in the control group (*P* < 0.05). The levels of glycosylated hemoglobin and total cholesterol in the control group were higher than those in the CHD group (*P* < 0.05), and the difference was statistically significant. No significant difference in other indices were detected (*P* > 0.05, Table 1).

**Table 1:**
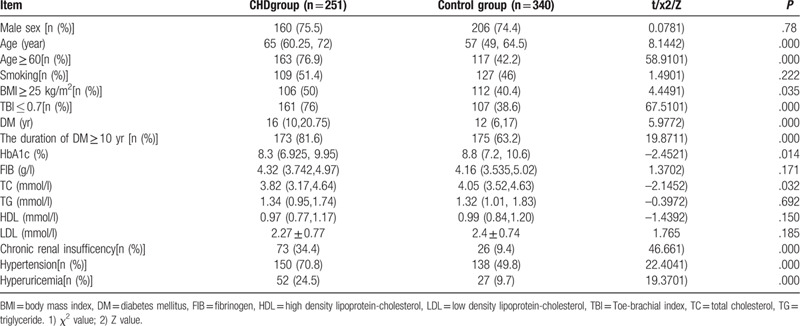
Comparison of baseline data between the 2 groups.

### 3.2 Risk factor analysis

Age (<60 years old = 0, ≥60 years old = 1), gender (female = 0, male = 1), BMI (<25 kg/m^2^ = 0, ≥25 kg/m^2^ = 1), duration of diabetes (<10 years = 0, ≥10 years = 1), TBI (>0.7 = 0, 0.7 = 1), chronic renal insufficiency (absent = 0, present = 1), hyperuricemia (absent = 0, present = 1), and hypertension (absent = 0, present = 1) were used as independent variables, and CHD (absent = 0, present = 1) was used as the dependent variable for logistic regression analysis. The results showed that age ≥60 years, duration of diabetes ≧10 years, TBI ≤ 0.7, chronic renal insufficiency, and hyperuricemia were significant risk factors for DF complicated with CHD (Table 2).

**Table 2:**
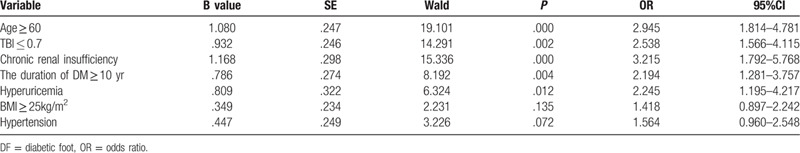
Logistic regression analysis of risk factors of DF complicated with coronary heart disease.

### 3.3 Establishment and evaluation of diagnostic model for DF complicated with CHD

#### 3.3.1 Establishment of the diagnostic model

According to OR value, the risk factor score was determined by the rounding-off principle. Age <60 years, TBI > 0.7, and normal renal function were recorded as 0 points, and age ≧60 years, TBI ≤ 0.7, and chronic renal dysfunction were recorded as 3 points. The duration of diabetes <10 years and absence of hyperuricemia were scored 0. Diabetes of 10 years and hyperuricemia: were scored 2 points. The total score is 13.

#### 3.3.2 Determination of diagnostic point and evaluation of diagnostic value of diagnostic model (Fig. 1)

When the diagnostic point was 5.5, the sensitivity and specificity were 74.5% and 70.4%, respectively. When the diagnostic point was 6.5, the sensitivity was 69.3% and the specificity was 76.5%, so the diagnostic point of CHD was 6 points. The area under the ROC curve of the diagnostic scoring system was 0.798 (0.759–0.837), and the standard error was 0.02. The diagnostic accuracy of the model was moderate and had application value.

**Figure 1:**
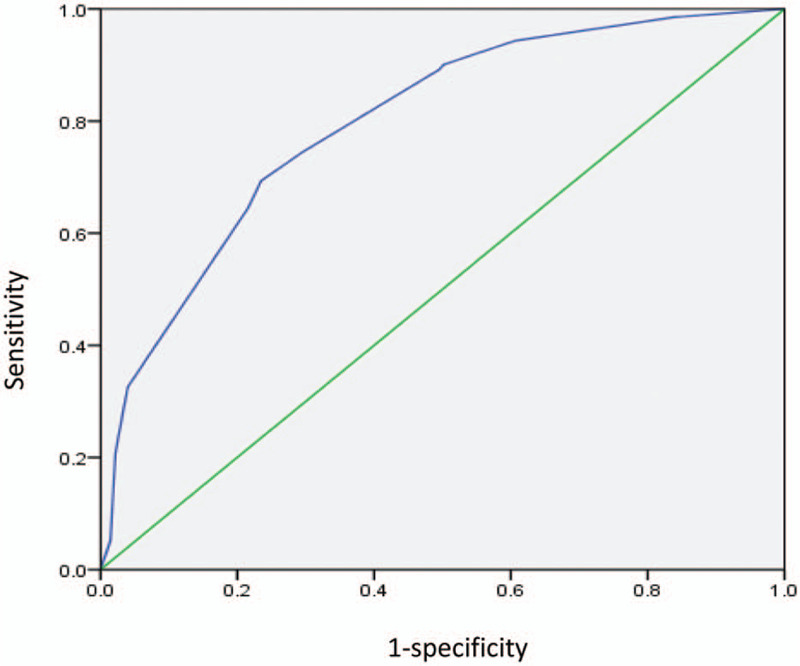
Diagnosis model ROC curve.

## 4 Discussion

DF is one of the serious complications of diabetes, and about 10% to 15% of diabetics developed DF in their lifetime.[^17^,^18^] DF is considered a marker of a significant increase in cardiovascular mortality.[^3^,^19^] Therefore, the diagnostic model of DF complicated with CHD established in this study can provide a simple and rapid method for diagnosis of patients with DF and CHD.

In this study, univariate and multivariate logistic regression analyses were used to screen and analyze the risk factors of patients with DF and CHD. The OR value was assigned to each risk factor, and the total score was 13 points. The diagnostic point of CHD was set according to the ROC curve, and the threshold value was 6 points. Patients with score ≥6 have a high- risk of developing CHD, and those with score <6 have a low- risk. High attention should be paid on to high-risk groups in clinical practice. Coronary angiography or CT examination of coronary arteries should be improved to evaluate the situation of coronary arteries, and relevant treatment should be given as soon as possible. For low-risk groups, regular follow-up should be conducted according to clinical symptoms.

In 2015, Meng-liwei et al established a diagnostic scoring system for CHD in patients with DF,^[20]^ and employed a statistical method for analysis. New patients with DF were selected, and the sample size was expanded to establish the diagnostic model of DF with CHD. According to the data, the area under the ROC curve of this study was 0.798, with a sensitivity of 69.3% and a specificity of 76.5%. Compared with the ROC curve area of 0.758, a sensitivity of 61.9% and a specificity of 75.5% were reported in the study of Meng-liwei et al. The diagnostic accuracy, sensitivity, and specificity of the present study were higher. The 2 studies selected five different risk factors and two identical risk factors. First of all, this study removed 2 risk factors which are gender and overweight. The reason was considered to be related to the expansion of sample size, the reduction of gender and BMI differences, and the closer proportion of patients in the disease group and the control group. Furthermore, overweight in diabetic patients is not more obvious in patients with CHD. Second, age stratification was inconsistent. The age span of the present study was larger (31–88 years old), with an average age of 60.71 years. The age span in the previous study was 40 to 87 years old, with an average age of 64.45 years, suggesting the possibility of early onset of DF. Third, ABI was used as the evaluation index for the stenosis of lower limb arteries in the previous study, while TBI 0.7 was used as the threshold to distinguish the presence of significant stenosis of lower limb arteries in the present study. ABI is widely used for initial evaluation of the recommended lower extremity perfusion. However, ABI is less sensitive to atherosclerosis in diabetic patients. Limitations on the predictive value of ABI in diabetic patients can be overcome by measuring TBI.[^21^,^22^,^23^,^24^] Fourth, hyperuricemia is added as a risk factor. Uric acid can play a pro-oxidative role, and oxidative stress can promote endothelial dysfunction. Hyperuricemia will cause damage to vascular endothelium and vascular wall.[^25^,^26^] Several studies have shown that hyperuricemia is associated and an independent risk factor for CHD.[^27^,^28^,^29^] In addition, the same risk factors in the 2 studies included diabetes history of 10 years and renal insufficiency, which were considered to be related to the long course of diabetes combined with macrovascular and microvascular diseases.

In summary, this study analyzes the risk factors for DF with CHD. Patients aged over 60 years, and have diabetes for 10 years or more, renal insufficiency, and lower limb vascular stenosis of DF should be given importance to screening of CHD. The diagnostic model can be a relatively accurate assessment tool for patients with DF and CHD. The model is a noninvasive, simple, and practical evaluation tool for diagnosis of CHD. Early intervention is recommended to prevent or reduce the occurrence of cardiovascular events in high-risk groups.

This research has some limitations, such as the condition of study population of the hospitalized patients, and high rates of diabetes complications, which may not be able to fully reflect the clinical features of patients in different stages of DF. Future studies should conduct multicenter, multi-channel, investigation to determine the efficiency and validate the diagnostic model to further improve its accuracy.

## Author contributions

**Conceptualization:** Caizhe Yang, Liwei Meng.

**Data curation:** Caizhe Yang, Ying Chen, Liangchen Wang.

**Formal analysis:** Caizhe Yang, Ying Chen, Liangchen Wang.

**Funding acquisition:** Caizhe Yang.

**Investigation:** Caizhe Yang, Ying Chen, Liwei Meng.

**Methodology:** Caizhe Yang, Ying Chen, Liwei Meng, Li Xiao.

**Project administration:** Ying Chen, Li Xiao.

**Resources:** Ying Chen, Li Xiao.

**Writing – original draft:** Ying Chen.

**Writing – review & editing:** Ying Chen.
